# Physical accessibility and utilization of health services in Yemen

**DOI:** 10.1186/1476-072X-9-38

**Published:** 2010-07-21

**Authors:** Abdullah Al-Taiar, Allan Clark, Joseph C Longenecker, Christopher JM Whitty

**Affiliations:** 1 Faculty of Medicine, Kuwait University, Box: 24923 Safat, 13110 Kuwait; 2 School of Medicine, Health Policy and Practice, University of East Anglia, Norwich, NR4 7TJ, UK; 3 London School of Hygiene and Tropical Medicine, Keppel St, London WC1E 6NL, UK; 4 Dept. Community Medicine and Behavioural Science, Faculty of Medicine, Kuwait University, Box: 24923 Safat, 13110 Kuwait

## Abstract

**Background:**

Assessment of physical access to health services is extremely important for planning. Complex methods that incorporate data inputs from road networks and transport systems are used to assess physical access to healthcare in industrialised countries. However, such data inputs hardly exist in many developing countries. Straight-line distances between the service provider and resident population are easily obtained but their relationship with driving distance and travel time is unclear. This study aimed to investigate the relationship between different measures of physical access, including straight-line distances, road distances and travel time and the impact of these measures on the vaccination of children in Yemen.

**Methods:**

Coordinates of houses and health facilities were determined by GPS machine in Urban and rural areas in Taiz province, Yemen. Road distances were measured by an odometer of a vehicle driven from participants' houses to the nearest health centre. Driving time was measured using a stop-watch. Data on children's vaccination were collected by personal interview and verified by inspecting vaccination cards.

**Results:**

There was a strong correlation between straight-line distances, driving distances and driving time (straight line distances vs. driving distance r = 0.92, p < 0.001, straight line distances vs. driving time r = 0.75; p < 0.001, driving distance vs. driving time r = 0.83, p < 0.001). Each measure of physical accessibility showed strong association with vaccination of children after adjusting for socio-economic status.

**Conclusion:**

Straight-line distances, driving distances and driving time are strongly linked and associated with vaccination uptake. Straight-line distances can be used to assess physical access to health services where data inputs on road networks and transport are lacking. Impact of physical access is clear in Yemen, highlighting the need for efforts to target vaccination and other preventive healthcare measures to children who live away from health facilities.

## Background

Access to health services is difficult to define. It is a multidimensional process that in addition to the quality of care, involves geographical accessibility, availability of the right type of care for those who need it, financial accessibility, and acceptability of service [1]. Geographic accessibility, the distance that must be traveled in order to use health facility, may present an important barrier of access to health services. Studies in developing countries have presented strong evidence that physical proximity of health service can play an important role in the use of primary healthcare [2-12]. In Yemen, we have demonstrated that driving distance and driving time are important predictors for developing severe malaria in comparison to mild malaria [13]. It is hypothesized that long distance can be a significant obstacle to reaching health facilities, and a disincentive even to trying to seek care [14].

The recent advances of Geographic Information Systems (GIS) have provided an important tool for healthcare planning particularly in measuring access to health services. Major progress was made in industrialized countries where the detailed data inputs such as detailed road network are available [15]. For example, Brabyn and Skelly used cost path analysis in order to determine the minimum travel time and distance to the closest hospital via road network in New Zealand [16]. More recently there was an attempt to produce a single index for the overall access to health services from combined physical access to the resources and the amount of resources available [17]. Application of such methods in developing countries, however, remained constrained by the lack of data inputs even in a hard copy form [15]. In developing countries roads are unpaved and adopted by convenience for travelling on foot or by vehicle. There is no well-established and functioning public transport system in many areas in developing countries. Instead measuring access to health services in developing countries remains imprecise and relies mostly on asking patients about the time and distance they travelled although most patients are not accustomed to watches. Additionally, acute (emergency) and preventative medical services are often taken together, which risks conflating two different challenges where physical barriers to care are different.

Travel impedance to the nearest health service provider is commonly used to assess the physical access to health services [18]. This is usually measured using Euclidean (straight-line) distance, driving distance or travel time. Driving distance and travel time is difficult to obtain in countries where there is a lack of well-established transport system or developed road networks. Straight-line distances are easily obtained but the relationship between straight-line distance and driving distance or travel time is not clear [19,20].

This study had two aims; firstly, to investigate whether the impact of distance on vaccination-an indicator of primary preventive healthcare use-is comparable with those found elsewhere. Secondly, to investigate the relationships between different measures of geographical access, namely straight-line distances, driving distances, and driving time. We aimed to assess the correlation between straight-line distances and both driving distance and driving time measured by a journey conducted by a research team and link this to the vaccination of a group of children in Yemen.

## Methods

### Study area and health facilities

Yemen is the most populous and poorest country in the Arabian Peninsula. Access to health services is problematic because of the vast geographical area and the sparse population distribution across the rural areas in addition to a poorly developed road networks and lack of proper public transport. Over 70% of Yemen's population of (19.7 million) live in rural areas (Population Census, 2004) where primary healthcare covers about 30% of the population (WHO, 2003). The vaccination coverage is estimated to be around 70%.

The study area was Taiz province, a densely populated region with 2.4 million residents (Census, 2004). The majority of people are casual labour workers on daily wages or subsistence farmers producing cereals. In urban and semi-urban areas people are engaged in small-scale trading activities in farm products or imported fabrics or work as shopkeepers. A few people work as civil servants, who are mostly teachers. There is no state-regulated public transport in the study area and the ownership of a car is low. Most people share a hired car in order to reach health facilities. Some car owners work in transport, with the frequency of their service depending on the number of passengers. There is no regular transport services and the cost of the same journey varies widely by the time of travel, availability of other passengers and many other factors. Most roads in the study area are locally adopted and are not recognized officially as roads.

Vaccination of children is available free of charge in all public health facilities. Private health services are available in urban and semi urban areas but most do not provide vaccination.

### Study subjects

Data were collected within two case-control studies on severe and mild malaria among children in Yemen within an area with radius of 40 km from the main referral hospital in Taiz. Details of the studies have been published elsewhere [13,21]. Briefly study subjects comprised three groups of children who were between 6 months and 10 years of age. The first group (children with severe malaria) was recruited from Yemeni Swedish referral hospital and traced to their homes. The second group (children with mild malaria) were recruited from several health centers in the study area and also traced to their homes. The third group (community controls without malaria) was selected from the neighborhood of the mild malaria cases using a random selection procedure.

### Data collection

Data on socio-economic factors were collected by face-to-face interviews combined with direct inspection during home visits. Driving distance between the households and health centers was measured by noting the distance shown on the odometer of a vehicle driving from the house to the nearest health centre for all the three groups. On a few occasions where there was more than one road, parents recommended the shortest and most convenient road they usually use to get access to health centre. Driving time of these journeys was measured using a stopwatch. GPS coordinates for houses and health facilities were taken by GPS (eTrex Summit^®^) by standing in front of the door of each household or health facility to achieve an accuracy goal of less than 15 meters.

A positive history of vaccination was defined as having been to a health facility to vaccinate the index child at least once. History of vaccination of the child was taken from the parents, and the child's vaccination card additionally was inspected during home visits.

### Statistical Methods

Data were double-entered in EPI Info version 6.04 (Centres for Disease Control and Prevention, Altlanta, USA) and analysed using Stata 8 (Stata Corporation, College Station, TX, USA).

The associations between different measurements of access were presented graphically as scatter plots. In order to calculate Pearson's correlation coefficients straight-line distance, driving distance, driving time were all log transformed because they were not normally distributed. The Kruskal-Wallis test was used to test for the differences in medians when the data were not normally distributed.

Unconditional logistic regression with vaccination status as the binary outcome variable, was used to adjust for socio-economic status in order to investigate the impact of each straight-line distance, driving distances or driving time on the vaccination of children. The likelihood ratio test was used to test for the significance of accessibility variables comparing models with and without the variable.

The straight-line distances were calculated using the Haversine formula which assumes that the earth is a perfect sphere and has radius of 6378.1 km [22]. This is a standard approach for the calculation of straight-line distances with GPS data, since the simple Pythogrean formula is generally not recommended. Straight-line distance, driving distance and driving time were categorized into quartiles as there are no well known cutoff points.

A socio-economic index was created based on 12 proxy indicators of socio-economic status. One score for deprivation was given for each of the following: Mother has not been to a school (or mother is dead); father has not been to a school (or father is dead); father's occupation (farmer, labourer, not working, or dead);, home with only one room, and absence of latrine, fridge, TV, radio, washing machine, telephone, vehicle and electricity. The sum of the scores was categorized into tertiles groups, least poor (0-6 score), middle poor (7-8 score), and most poor (9-12 score).

## Results

At the end of the study 334 severe malaria cases, 437 mild malaria cases and 308 community controls were recruited (17 could not be traced and were excluded from this analysis). Another 35 had missing or erroneous GPS coordinates. This analysis therefore included 1044 subjects some of whom came from the same household. Of all subjects, 80.7% were from rural areas while 10% from semi-urban areas and the rest from urban areas.

### Relationship between different measures of physical access

The median driving distance to the nearest governmental centre was 7.0 km (IQR: 2.0-12.0). The median straight-line distance was 2.4 km (IQR: 1.0-4.3). The proportion of children within 5 km driving distance was 44.6%, compared to 82.6% within 5 km measured by straight-line distances.

**Figure 1:**
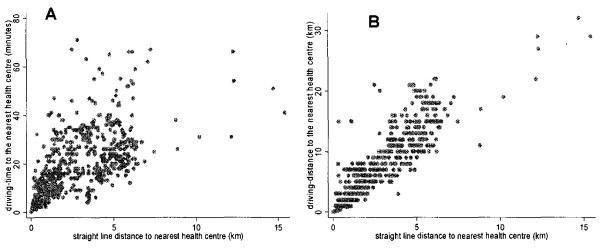
**The relationship between straight line distances and driving distance and driving time**.

**Figure 2:**
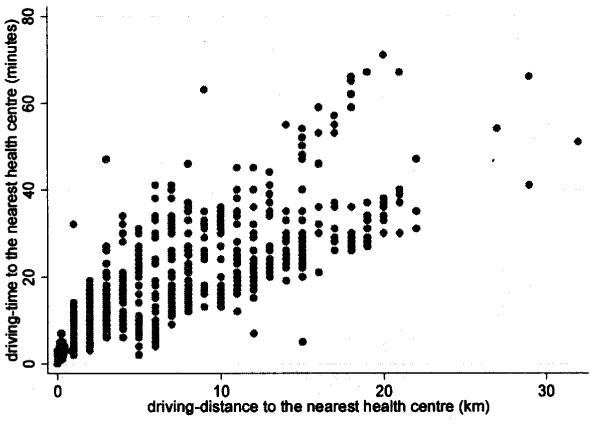
**The relationship between driving distance and driving time**.

The association between the driving distances and the straight-line distances, to the nearest health centre is presented in Figure 1. a. There was a strong correlation between the driving distance and the straight-line distances to the nearest health centres, correlation coefficient 0.92 (p < 0.001). The relationship between the straight-line distances and the driving time to health centres is illustrated in Figure 1. b (correlation coefficient, r = 0.75; p < 0.001). The association between the driving distance and the driving time to the health centre is shown in Figure 2, with a correlation coefficient of 0.83 (p < 0.001).

### Association between the different measures of physical access and vaccination of the children

Out of 1044 children, 726 (69.5%) reported they had been to a health facility to vaccinate their child on at least one occasion. The history of vaccination was validated by reviewing the child's vaccination card during home visit. Although 533 (48.8%) said they have the vaccination card, only 209/1044 (20.0%) were able to find the vaccination card during the home visit. It was also apparent that the older the child, the less likely it was that the parents could find the vaccination card.

The median driving distance was 6.0 km (IQR: 2.0-12.0) among vaccinated children and 8.0 km (IQR: 3.0-13.0) among non-vaccinated children (p < 0.001). Similarly, the median driving time was 16.0 minutes (IQR: 9.0-28.0) and 21.0 minutes (IQR: 11.0-29.0) among vaccinated and non-vaccinated children, respectively (p < 0.001). The differences in the driving distance and time between vaccinated and non-vaccinated were evident in all groups (severe malaria, mild malaria and community controls). Similarly, the median for the straight-line distances was 2.0 km (IQR: 0.9-4) and 2.7 km (IQR: 1.2-4.9) among vaccinated and non-vaccinated children respectively (p = 0.004). Straight-line distance, driving distance and driving time when categorized into groups showed highly significant positive crude associations with the history of vaccination of children, (χ^2 ^for linear trend p = 0.001, p = 0.008, p < 0.001 respectively), Table 1.

**Table 1:** Proportion of children vaccinated by different measures of physical access to health services in Yemen.

Measure of physical access	Vaccination		Total
**Straight-line Distance n(%)**	Yes	No	

Zero-	216 (78.3)	60 (21.7)	276

1 km-	171 (64.8)	93 (35.2)	264

2.5 Km-	190 (72.0)	74 (28.0)	264

4.5 Km-	149 (62.1)	91 (37.9)	240

χ^2 ^for linear trend = 10.4, p = 0.001			

**Driving Distance n(%)**			

Zero-	132 (79.0)	35 (21.0)	167

2 km-	248 (71.9)	97 (28.1)	345

7 Km-	147 (60.2)	97 (39.8)	244

12 Km-	199 (69.1)	89 (30.9)	288

χ^2 ^for linear trend = 6.9, p = 0.009			

**Driving Distance n(%)**			

Zero-	199 (80.6)	48 (19.4)	247

10 Minutes-	169 (69.0)	76 (31.0)	245

17 Minutes-	172 (61.9)	106 (38.1)	278

29 Minutes-	186 (67.9)	88 (32.1)	274

χ^2 ^for linear trend = 12.0, p < 0.001			

Table 2 shows the association between each measure of physical access and the vaccination of the children before and after controlling for socio-economic status and age of the child. There was a strong association between vaccination of children and straight-line distance before and after adjusting for age of the child and socio-economic status. Similarly, both driving distance and driving time showed significant association with the vaccination of children in univariate and multivariate analysis.

**Table 2:** Association between different measures of access and vaccination of children before and after adjusting for socio-economic status and age of the child (1 = not vaccinated, 0 = vaccinated)

Measures of access	Crude odds ratio (95%CI)	p-value	Adjusted odds ratio (95%CI)	p-value
**Area of residence**				
Urban	1(Reference)	0.04		
Semi-urban	2.75(0.77-9.8)			
Rural	3.50(1.18-10.43)			

**Socio-economic status**				
Least poor	1(Reference)	<0.001		
Middle poor	2.22(1.47-3.56)			
Most poor	3.80(2.63-5.50)			

**Straight-line distance (km)**				
Zero-	1(Reference)	<0.001	1(Reference)	0.001
1 km-	1.96(1.34-2.87)		2.02(1.36-3.00)	
2.5 km-	1.40(0.95-2.08)		1.49(0.99-2.23)	
4.5 km-	2.20(1.49-3.24)		1.92(1.29-2.86)	

**Driving distance (km)**				
Zero-	1(Reference)	<0.001	1(Reference)	0.005
2 km-	1.48(0.95-2.29)		1.41(0.90-2.22)	
7 km-	2.49(1.58-3.91)		2.24(1.40-3.57)	
12 km-	1.69(1.08-2.64)		1.56(0.98-2.47)	

**Driving time (minutes)**				
Zero-	1(Reference)	<0.001	1(Reference)	0.003
10 minutes-	1.86(1.23-2.82)		1.52(0.99-2.34)	
17 minutes-	2.55(1.72-3.80)		2.14(1.42-3.23)	
29 minutes-	1.96(1.30-2.94)		1.75(1.15-2.65)	

## Discussion

Measuring geographical access to health services using self-reported data is not reliable because it is difficult for patients to remember the distance they have traveled or the time of the journey [9]. Instead physical access to the nearest health service is usually estimated by straight-line distance, driving distance or travel time [18]. Driving distance and travel time is difficult to obtain where there is no transport system or well-developed road networks. Straight-line distances are easily obtained but its relationship with driving distance or travel time is not always clear. This study aimed to investigate the relationship between different measures of distance to health services in Yemen and to determine the association between these measures and utilization of preventive health services. It demonstrates that straight-line distance is strongly linked to driving distance and travel time. Straight-line distance, like driving distance and driving time, is an independent predictor for vaccination of children, a reasonable proxy for preventative medical services.

Using GIS technology is recommend to achieve more effective and efficient planning for primary healthcare [23]. In industrialized countries presence of digital road maps, well-established public transport, information on the residence of the population and on the resources available in each health facility were all combined to create indicators for the accessibility to health services. However, much of these data inputs are not available in many developing countries even in hard copy form [15]. One aspect of accessibility to health services that is usually overlooked [24], is the distance which must be travelled in order to use health services. This is usually estimated by straight-line distance or with more complexity network distance that include road distance and travel time. Straight-line distance are easy to calculate and compare and can be used in settings where data inputs on roads or transport are lacking. To which extent straight-line distance reflects the driving distance and travel time in different settings is not clear. Jordan et al. found that straight-line distances are closely correlated with the more complex drive-times in South West England although low correlation was concentrated in peripheral areas [19]. In our study, straight-line distances showed significant correlation with driving distance and driving time (r = 0.92 and r = 0.75 respectively). More importantly, it is an independent predictor for vaccination of children suggesting that this less complex measure can be used to estimate physical access in our setting and similar settings. The correlation between driving distances and driving time was marginally stronger than correlation between straight-line distances and driving time (r = 0.83 Vs r = 0.75). This is plausible as driving time is not usually linearly linked to distance because of the topography, and in urban settings one way streets or traffic.

Vaccination is considered the most cost-effective preventive intervention for children [25-27]. Our findings showed strong associations between vaccination of children and distance whether estimated as straight-line distance, driving distance, or driving time. In Pakistan neither driving distance nor driving time showed association with the use of public health services for treatment of acute childhood illness [28]. Similarly no association was found between the use of contraceptive services and straight-line distances in Malawi [29]. In Kenya, however, straight-line distances showed significant association with utilization of health services [11]. It is not clear whether this difference reflects differences between settings, or between preventative and curative medical services-but it is probably both.

In Yemen, we have previously demonstrated that driving distance and driving time are independent predictors for developing severe malaria in comparison to mild malaria [13]. In this current work, distance seems to be an important predictor for using preventive health services like vaccination. Unlike the situation in Pakistan [28], Nigeria [30] or Malawi [29] where the distance has less profound impact on utilization of health services, distance seems to have a strong impact on utilization of preventive health services in Yemen. New health facilities providing preventative services should be located where they would serve most people, yet minimize the distance to be traveled. Straight-line distance which is a less complex measure of physical access can guide this as it is strongly linked to driving distance and driving time and also an independent predictor for utilization. This, however, does not imply that shortening the distance will quickly fix the problem of utilization of health services in the country.

One of the limitations of this study is the reliability of the vaccination history, as the information about vaccination status obtained from parents is less reliable than those from child health cards [31]. The older the child is, the more inaccurate is the information mothers provide [32]. As only a limited number of families were able to provide the vaccination card during home visits we could not ascertain the number of children who were fully vaccinated, although it has been reported that access to healthcare influences the frequency of service use [33-35].

We have also made several assumptions, including that the three study groups are a reasonably representative sample of the population in the study area, that parents had used the nearest health facility to vaccinate their child, and that the family's socio-economic status has not changed since the vaccination of the child.

## Conclusion

We have demonstrated that straight-line distances are strongly correlated with driving distance and driving time in Yemen. It is also an independent predictor for vaccination of children. As a measure of physical access, straight-line distances are easily obtained in comparison to travel time and travel distance which require detailed data inputs on road networks and transport that hardly exist in Yemen or similar settings. Our findings suggest that straight-line distances have potential to be used in planning of health services in countries such as Yemen with limited information and with difficult terrain. The impact of physical access on using health services in Yemen seems to be far more clear than in some other settings. Therefore, greater efforts need to be made to provide at least basic preventive services (e.g. vaccination) to children who live far from primary healthcare centres.

## Competing interests

The authors declare that they have no competing interests.

## Authors' contributions

AAT: contributed to conception and design of the study, acquisition of the data, analysis and interpretation of the data, and drafting the manuscript. AC: contributed to analysis and interpretation of the data, and critical revision of the manuscript for important intellectual content. JCL: contributed to analysis and interpretation of the data, and critical revision of the manuscript for important intellectual content. CW: contributed to conception and design, and critical revision of the manuscript for important intellectual content. All authors read and approved the final manuscript.

## References

[B1] Peters DH Garg A Bloom G Walker DG Brieger WR Rahman MH Poverty and access to health care in developing countries Ann N Y Acad Sci 2008 1136 161 171 10.1196/annals.1425.011 17954679

[B2] Bashshur RL Shannon GW Metzner CA Some ecological differentials in the use of medical services Health Serv Res 1971 6 61 75 5569227 PMC1067311

[B3] Stock R Distance and the utilization of health facilities in rural Nigeria Soc Sci Med 1983 17 563 570 10.1016/0277-9536(83)90298-8 6879255

[B4] Nnadi EE Kabat HF Choosing health care services in Nigeria: a developing nation J Trop Med Hyg 1984 87 47 51 6748128

[B5] Abbas AA Walker GJ Determinants of the utilization of maternal and child health services in Jordan Int J Epidemiol 1986 15 404 407 10.1093/ije/15.3.404 3771079

[B6] Kloos H Utilization of selected hospitals, health centres and health stations in central, southern and western Ethiopia Soc Sci Med 1990 31 101 114 10.1016/0277-9536(90)90052-T 2389147

[B7] Paul BK Health service resources as determinants of infant death in rural Bangladesh: an empirical study Soc Sci Med 1991 32 43 49 10.1016/0277-9536(91)90125-V 2008620

[B8] Airey T The impact of road construction on the spatial characteristics of hospital utilization in the Meru district of Kenya Soc Sci Med 1992 34 1135 1146 10.1016/0277-9536(92)90287-Z 1641675

[B9] Muller I Smith T Mellor S Rare L Genton B The effect of distance from home on attendance at a small rural health centre in Papua New Guinea Int J Epidemiol 1998 27 878 884 10.1093/ije/27.5.878 9839747

[B10] Buor D Analysing the primacy of distance in the utilization of health services in the Ahafo-Ano South district, Ghana Int J Health Plann Manage 2003 18 293 311 10.1002/hpm.729 14727709

[B11] Noor AM Zurovac D Hay SI Ochola SA Snow RW Defining equity in physical access to clinical services using geographical information systems as part of malaria planning and monitoring in Kenya Trop Med Int Health 2003 8 917 926 10.1046/j.1365-3156.2003.01112.x 14516303 PMC2912492

[B12] Feikin DR Nguyen LM Adazu K Ombok M Audi A Slutsker L Lindblade KA The impact of distance of residence from a peripheral health facility on pediatric health utilisation in rural western Kenya Trop Med Int Health 2009 14 54 61 10.1111/j.1365-3156.2008.02193.x 19021892

[B13] Al-Taiar A Jaffar S Assabri A Al-Habori M Azazy A Al-Gabri A Al-Ganadi M Attal B Whitty CJ Who develops severe malaria? Impact of access to healthcare, socio-economic and environmental factors on children in Yemen: a case-control study Trop Med Int Health 2008 13 762 770 18410250 10.1111/j.1365-3156.2008.02066.x

[B14] Rahaman MM Aziz KM Munshi MH Patwari Y Rahman M A diarrhea clinic in rural Bangladesh: influence of distance, age, and sex on attendance and diarrheal mortality Am J Public Health 1982 72 1124 1128 10.2105/AJPH.72.10.1124 7114335 PMC1650188

[B15] Perry B Gesler W Physical access to primary health care in Andean Bolivia Soc Sci Med 2000 50 1177 1188 10.1016/S0277-9536(99)00364-0 10728839

[B16] Brabyn L Skelly C Modeling population access to New Zealand public hospitals Int J Health Geogr 2002 1 3 10.1186/1476-072X-1-3 12459048 PMC149398

[B17] Ray N Ebener S AccessMod 3.0: computing geographic coverage and accessibility to health care services using anisotropic movement of patients Int J Health Geogr 2008 7 63 10.1186/1476-072X-7-63 19087277 PMC2651127

[B18] Guagliardo MF Spatial accessibility of primary care: concepts, methods and challenges Int J Health Geogr 2004 3 3 10.1186/1476-072X-3-3 14987337 PMC394340

[B19] Jordan H Roderick P Martin D Barnett S Distance, rurality and the need for care: access to health services in South West England Int J Health Geogr 2004 3 21 10.1186/1476-072X-3-21 15456514 PMC524184

[B20] Apparicio P Abdelmajid M Riva M Shearmur R Comparing alternative approaches to measuring the geographical accessibility of urban health services: Distance types and aggregation-error issues Int J Health Geogr 2008 7 7 10.1186/1476-072X-7-7 18282284 PMC2265683

[B21] Al-Taiar A Assabri A Al-Habori M Azazy A Algabri A Alganadi M Whitty CJ Jaffar S Socioeconomic and environmental factors important for acquiring non-severe malaria in children in Yemen: a case-control study Trans R Soc Trop Med Hyg 2009 103 72 78 10.1016/j.trstmh.2008.09.010 18950826

[B22] Sinnott R Virtues of the Haversine Sky and Telescope 1984 68 159

[B23] Brabyn L Barnett R Population need and geographical access to general practitioners in rural New Zealand N Z Med J 2004 117 U996 15475979

[B24] Goddard M Smith P Equity of access to health care services: theory and evidence from the UK Soc Sci Med 2001 53 1149 1162 10.1016/S0277-9536(00)00415-9 11556606

[B25] Zhou F Santoli J Messonnier ML Yusuf HR Shefer A Chu SY Rodewald L Harpaz R Economic evaluation of the 7-vaccine routine childhood immunization schedule in the United States, 2001 Arch Pediatr Adolesc Med 2005 159 1136 1144 10.1001/archpedi.159.12.1136 16330737

[B26] Dayan GH Cairns L Sangrujee N Mtonga A Nguyen V Strebel P Cost-effectiveness of three different vaccination strategies against measles in Zambian children Vaccine 2004 22 475 484 10.1016/j.vaccine.2003.07.007 14670330

[B27] Acharya A Diaz-Ortega JL Tambini G de Quadros C Arita I Cost-effectiveness of measles elimination in Latin America and the Caribbean: a prospective analysis Vaccine 2002 20 3332 3341 10.1016/S0264-410X(02)00296-7 12213403

[B28] NoorAli R Luby S Rahbar MH Does use of a government service depend on distance from the health facility? Health Policy Plan 1999 14 191 197 10.1093/heapol/14.2.191 10538722

[B29] Heard NJ Larsen U Hozumi D Investigating access to reproductive health services using GIS: proximity to services and the use of modern contraceptives in Malawi Afr J Reprod Health 2004 8 164 179 10.2307/3583189 15623130

[B30] Mburu FM Smith MC Sharpe TR The determinations of health services utilization in a rural community in Kenya Soc Sci Med 1978 12 211 217 10.1016/S0277-9536(78)80009-4 675273

[B31] Ramakrishnan R Rao TV Sundaramoorthy L Joshua V Magnitude of recall bias in the estimation of immunization coverage and its determinants Indian Pediatr 1999 36 881 885 10744865

[B32] Suarez L Simpson DM Smith DR Errors and correlates in parental recall of child immunizations: effects on vaccination coverage estimates Pediatrics 1997 99 E3 10.1542/peds.99.5.e3 9113960

[B33] Weiss JE Greenlick MR Determinants of medical care utilization: the effect of social class and distance on ontacts with the medical care system Med Care 1970 8 456 462 10.1097/00005650-197011000-00003 5489515

[B34] Shannon GW The concept of distance as a factor in accessibility and the utilisation of health care Medical Care Review 1969 6 143

[B35] Metzner CA The concept of distance as a factor in accessibility and the utilisation of health care Medical Care Review 1969 26 243 261

